# Analysis of stress response distribution in patients with lateral ankle ligament injuries: a study of neural control strategies utilizing predictive computing models

**DOI:** 10.3389/fphys.2024.1438194

**Published:** 2024-07-24

**Authors:** Zhifeng Zhou, Huiyu Zhou, Tianle Jie, Datao Xu, Ee-Chon Teo, Meizi Wang, Yaodong Gu

**Affiliations:** ^1^ Faculty of Sports Science, Ningbo University, Ningbo, China; ^2^ Faculty of Engineering, University of Pannonia, Veszprem, Hungary; ^3^ School of Mechanical and Aerospace Engineering, Nanyang Technological University, Singapore, Singapore; ^4^ Department of Biomedical Engineering, Faculty of Engineering, The Hong Kong Polytechnic University, Kowloon, Hong Kong SAR, China

**Keywords:** lateral ankle ligament injury, finite element analysis, foot, ligament mechanics, composite material

## Abstract

**Background:**

Ankle sprains are prevalent in sports, often causing complex injuries to the lateral ligaments. Among these, anterior talofibular ligament (ATFL) injuries constitute 85%, and calcaneofibular ligament (CFL) injuries comprise 35%. Despite conservative treatment, some ankle sprain patients develop chronic lateral ankle instability (CLAI). Thus, this study aimed to investigate stress response and neural control alterations during landing in lateral ankle ligament injury patients.

**Method:**

This study recruited twenty individuals from a Healthy group and twenty CLAI patients performed a landing task using relevant instruments to collect biomechanical data. The study constructed a finite element (FE) foot model to examine stress responses in the presence of laxity of the lateral ankle ligaments. The lateral ankle ligament was modeled as a hyperelastic composite structure with a refined representation of collagen bundles and ligament laxity was simulated by adjusting material parameters. Finally, the validity of the finite element model is verified by a high-speed dual fluoroscopic imaging system (DFIS).

**Result:**

CLAI patients exhibited earlier Vastus medialis (*p* < 0.001) and tibialis anterior (*p* < 0.001) muscle activation during landing. The FE analysis revealed that with laxity in the ATFL, the peak von Mises stress in the fifth metatarsal was 20.74 MPa, while with laxity in the CFL, it was 17.52 MPa. However, when both ligaments were relaxed simultaneously, the peak von Mises stress surged to 21.93 MPa. When the ATFL exhibits laxity, the CFL is subjected to a higher stress of 3.84 MPa. Conversely, when the CFL displays laxity, the ATFL experiences a peak von Mises stress of 2.34 MPa.

**Conclusion:**

This study found that changes in the laxity of the ATFL and the CFL are linked to shifts in metatarsal stress levels, potentially affecting ankle joint stability. These alterations may contribute to the progression towards CLAI in individuals with posterolateral ankle ligament injuries. Additionally, significant muscle activation pattern changes were observed in CLAI patients, suggesting altered neural control strategies post-ankle ligament injury.

## 1 Introduction

Lateral Ankle Ligament (LAL) injuries are among the most prevalent injuries affecting the ankle joint, with occurrences noted across various populations and levels of athletic activity (Swanson et al., 2022). According to research statistics, LAL injuries represent approximately 85% or more of all ankle injuries (Ferran and Maffulli, 2006). Among these, a prevalent type of LAL injury is the anterior talofibular ligament (ATFL) injury, often occurring concomitantly with injuries to the calcaneofibular ligament (CFL) or both the calcaneofibular ligament and posterior talofibular ligament (PTFL) (DiGiovanni et al., 2004; Maffulli et al., 2008). Injuries to the ATFL represent 85% of these injuries, while injuries to the CFL account for 35%. Extending from the anterior margin of the lateral fibular ankle to the neck of the talus, ATFL is also recognized as the structurally weakest among the lateral ankle ligaments (Khawaji and Soames, 2015). Despite a 60%–80% healing rate with conservative treatment for ankle sprains, cases where injuries fail to heal properly may lead to chronic lateral ankle instability (CLAI) (Hintermann et al., 2002). In this case, altered kinematics of the tibial talonavicular joint and increased cartilage contact stresses lead to degenerative joint disease (Bischof et al., 2010).

Injury to the LAL not only induces ligament structural alterations but may also exert diverse impacts on the neuromuscular system. Research indicates that following LAL injury, patients may experience diminished neuromuscular recruitment, weakened muscle strength, and impaired motor coordination (Gribble et al., 2013). These consequences can manifest as challenges in daily activities, including uncoordinated gait patterns during walking and running, or even difficulties in maintaining postural stability. Damage to the neuromuscular system not only impacts the patient’s quality of life but also directly influences their motor performance and athletic capabilities (Xu et al., 2023a). Diminished neuromuscular recruitment is a common consequence following LAL injury (Doherty et al., 2014). Ligament injuries can result in reduced joint proprioception, affecting the nervous system’s perception and responsiveness, consequently diminishing the sensitivity and responsiveness of muscles to nerve signals (Kunugi et al., 2017). This can decrease muscle activation, disrupting normal motor control and movement coordination. Moreover, LAL injuries may contribute to a decline in muscle strength. Because ligament injuries induce joint instability, the adjacent muscles may undergo functional deterioration, resulting in diminished muscle strength (Lin et al., 2019; Han et al., 2022). This reduction in muscle strength can exacerbate joint instability, perpetuating a cycle of vulnerability to injury (Xu et al., 2023b).

Studies have shown that the LAL plays a crucial role in maintaining ankle stability, and injury to it may lead to impaired joint stability (Gribble et al., 2016). Patients may experience ankle laxity and lateral instability following injury, increasing the risk of re-sprain or recurrence (Xu et al., 2024). Normally, the LAL maintains stability by supporting the ankle and limiting joint motion. However, once the LAL is damaged, the joint’s stability will be compromised. Secondly, the loss of ankle stability not only affects the patient’s daily activities but may also increase the risk of joint damage (Hintermann et al., 2004). Due to the lack of joint stability, patients are more susceptible to external impact or torque during sports or activities, increasing the likelihood of re-injury (Bae et al., 2015). Ankle instability may also damage the periarticular soft tissues, exacerbating joint dysfunction and pain symptoms. Chronic ankle instability can ultimately result in cartilage degeneration and the eventual development of ankle osteoarthritis (Valderrabano et al., 2006). Long-term follow-up studies have reported that osteoarthritis occurs in 13%–78% of patients with ankle instability over 10 years (Valderrabano et al., 2009). Additionally, recent studies involving patients have identified LAL injuries as the primary cause of ankle osteoarthritis following ligamentous lesions (Löfvenberg et al., 1994).

The finite element (FE) method plays an important role in the field of biomechanics, enabling in-depth analysis of the behavior of complex joints and tissues under clinically relevant loading conditions. It not only accurately simulates real-life scenarios but also provides insights beyond traditional biomechanical studies. When analyzing ankle stability, finite element modeling emerges as a powerful tool for studying the mechanical behavior of the lateral ligament of the ankle. The material behavior of both the ankle and the lateral collateral ligament typically exhibits nonlinearity and nonuniformity, particularly under conditions involving significant deformations and high strain rates (Telfer et al., 2014). Finite element models can integrate these material properties, thereby enabling a more precise simulation of the stress-strain response of the ligaments, as well as the deformation behavior under varying loading conditions (Peng et al., 2023). By meticulously modeling the geometry and material properties of the ankle joint, finite element simulations offer researchers valuable insights into the mechanisms governing ankle stability. Additionally, they unveil the internal stress distribution and deformation patterns of the ligaments under stress conditions (Mabrouk et al., 2022).

There have been previous finite element studies on the ankle joint. Mangwani et al. utilized finite elements to analyze the impacts of ankle ligament injuries, including lateral, syndesmotic, and medial injuries (Talbott et al., 2023). They also investigated the effects of stepwise repairs for each injury on joint displacements and contact stresses, providing valuable insights into optimal repair strategies and prognosis (Halloran et al., 2023). Furthermore, finite element modeling can optimize techniques such as determining the number and placement of bone anchors in lateral ligament reconstruction, evaluating the impact of fibrous bands or hamstring reinforcement on lateral ligament reconstruction, and dynamically stabilizing screws in the joint (Shin et al., 2012). It can also quantify the effects of bone alignment, such as heel pronation and correction. Additionally, finite element analysis can serve as a treatment and reattachment strategy for insertional Achilles tendinopathy (Tits and Ruffoni, 2021).

Previous research has not explored the changes in joint stability and stress response distribution in patients with ankle ligament injuries during landing, understanding these changes is crucial for developing effective rehabilitation strategies and preventive measures for ankle ligament injuries. Therefore, this study aimed to explore the stress response and neural control changes during landing in individuals with lateral ankle ligament injuries and to evaluate the impact of varying degrees of ligament laxity on metatarsal stress. Our hypothesis posited that metatarsal stress would escalate with increased ligament laxity.

## 2 Method

The main framework of this study consists of the following components: 1) Collection of subject biomechanical data. 2) Processing data using Matlab. 3) Construction of finite element models. 4) Validation of finite element model results using a high-speed dual fluoroscopic imaging system (DFIS). First, kinematic and kinetic data of the participants were collected using Kistler force plates and Vicon, while sEMG signals were gathered using EMG sensors. Next, the data were preprocessed using MATLAB. Then, a finite element model was constructed, and the data were imported. Finally, the results of the finite element model were validated using DFIS.

## 3 Subjects

Twenty healthy subjects and twenty subjects with CLAI were recruited for this study (Table 1). All CLAI subjects were treated conservatively for 6 months before the experiment; however, they continued to exhibit symptoms of pain, instability and decreased proprioception. The diagnosis of CLAI was confirmed through a clinical examination by a foot and ankle orthopedic surgeon, and the clinical manifestations of lateral ankle injury were confirmed by magnetic resonance imaging (MRI). The criteria for subject screening were: 1) no history of foot and ankle surgeries; 2) no additional ankle pathology was identified by MRI. Reasons for exclusion included peroneal tendinopathy, and intra-articular small bone or cartilage degeneration (Caputo et al., 2009).

**TABLE 1:** Participant demographics.

	CLAI (n = 20)	Healthy (n = 20)	P
Age (year)	23.8 ± 1.5	24.1 ± 1.3	0.441
Mass (kg)	82.3 ± 4.9	81.1 ± 6.1	0.738
Height (cm)	181.3 ± 3.2	179.6 ± 5.4	0.535
Ankle sprains (times)	3.4 ± 1.2	0	<0.001
Time since injury (month)	10.2 ± 2.3	0	<0.001
Leg length (cm)	95.7 ± 4.5	92.9 ± 5.2	0.769

CLAI, chronic lateral ankle instability.

Prior to engaging in the study, all individuals were thoroughly informed on the study’s purpose, requirements, and procedures. This information was provided after each participant had signed the informed consent form. The study protocol received approval from the Ningbo University Scientific Research Ethics Committee (Approval Number: RAGH20240405).

### 3.1 Biomechanics parameters collection and processing

**FIGURE 1:**
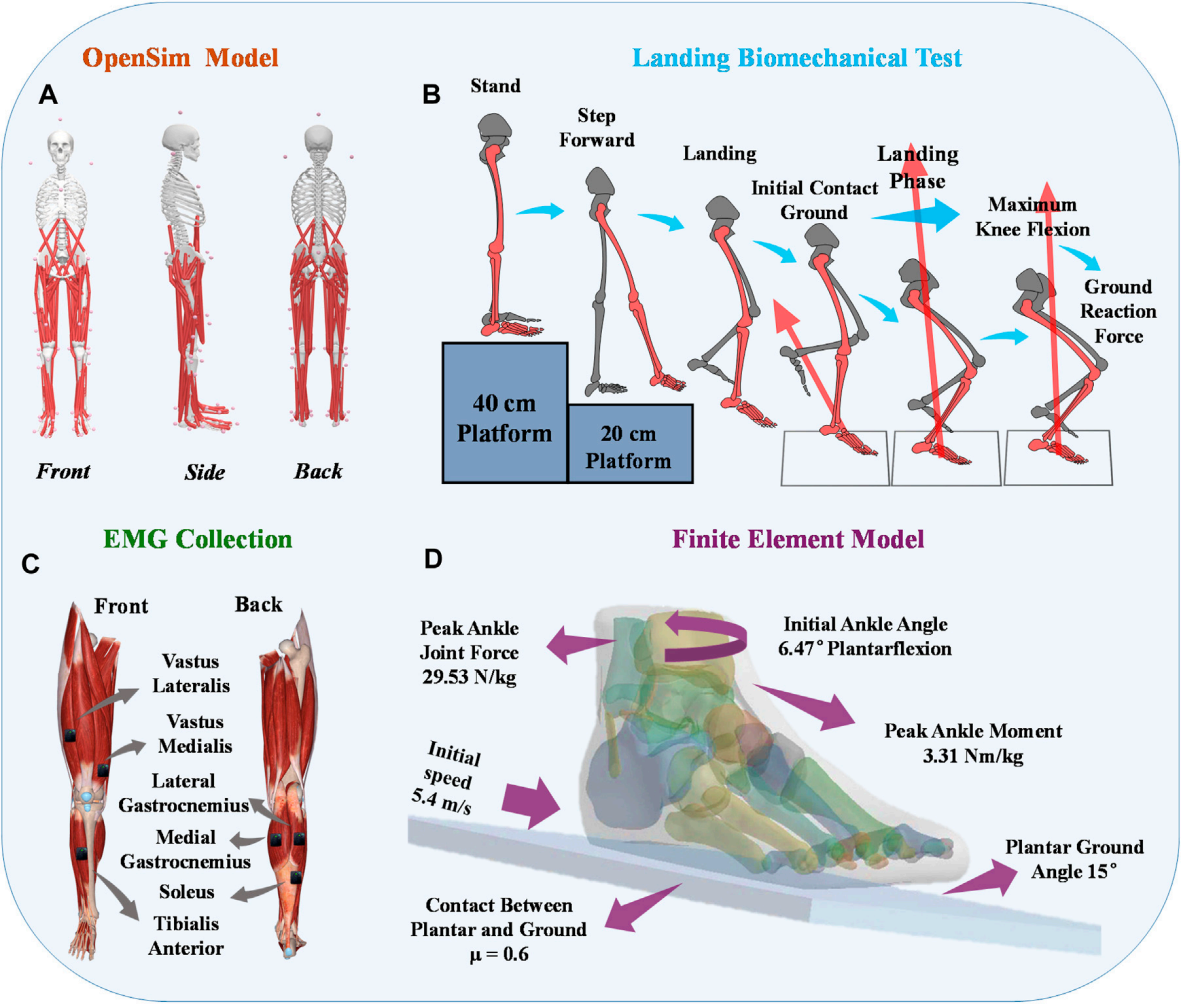
Illustrative representation depicting the placement of reflective marker points on the musculoskeletal model. **(A)** Visual representation of the placement of reflective markers on the constructed musculoskeletal model. **(B)** Depiction of the positioning of EMG electrodes on the lower limbs of human subjects. **(C)** Illustration outlining the procedure of the unanticipated landing biomechanics test. **(D)** Finite Element Boundary Condition Setting.

**FIGURE 2:**
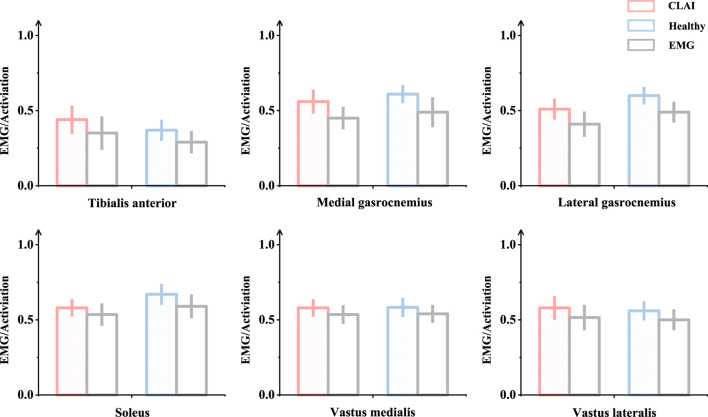
Validation was conducted by comparing the patterns of measured muscle activation and simulated activation.

This study utilized a Kistler force platform and an eight-camera Vicon motion capture system (Oxford Metrics Ltd., Oxford, United Kingdom) to synchronize the kinetic and kinematic data collection. Kinematic data collection was performed at frequencies of 100 Hz, while kinetic data collection was conducted at frequencies of 200 Hz (Ward et al., 2009). Markers were positioned according to the gait 2,392 (Figure 1A), and EMG sensors were placed following the guidelines provided by SENIAM (Figure 1C) (Hermens et al., 2000). This study minimized the impedance of the skin-electrode interface by shaving the hair near the skin and cleaning the area with alcohol. Muscle activation was measured using six electromyography (EMG) sensors from Delsys (Boston, MA, United States) (Figure 2). All subjects were instructed to perform landings from two consecutive steps to collect the corresponding kinetic data. Data was successfully collected 20 times for each subject, recording only the data from stable landings (Figure 1B). The three-dimensional marker trajectories and ground reaction force data were collected using Vicon Nexus 2.14.0 and exported as C3D format files. These data were then processed in MATLAB (MathWorks, MA, United States), which involved coordinate system conversion, low-pass filtering, data extraction, and format conversion for both the kinematic and ground reaction force data. This study utilized the OpenSim software (Stanford, CA, United States) for finite element analysis to calculate biomechanical parameters as the boundary condition (Cheung et al., 2009).

### 3.2 The process of obtaining and reconstructing geometric data

One of the subjects with chronic lateral ankle instability (CLAI) was chosen to undergo magnetic resonance imaging (MRI) and computed tomography (CT) with a 2 mm interval for this study. The resulting 2D images were then segmented using Mimics 21.0 (Materialise, Leuven, Belgium). Subsequently, the generated foot model was imported into Geomagic Studio 2021 (Geomagic, Inc., Research Triangle Park, NC, United States) to optimize the model. The imported components were then assembled into solid form using SolidWorks 2017 (SolidWorks Corporation, Waltham, MA, United States). Finally, the model’s contacts were meshed and modeled using Workbench 2021 (ANSYS, Inc. located in Canonsburg, PA, United States), and static finite element analysis was conducted (Figure 1D).

### 3.3 Composite representation of the LAL

**FIGURE 3:**
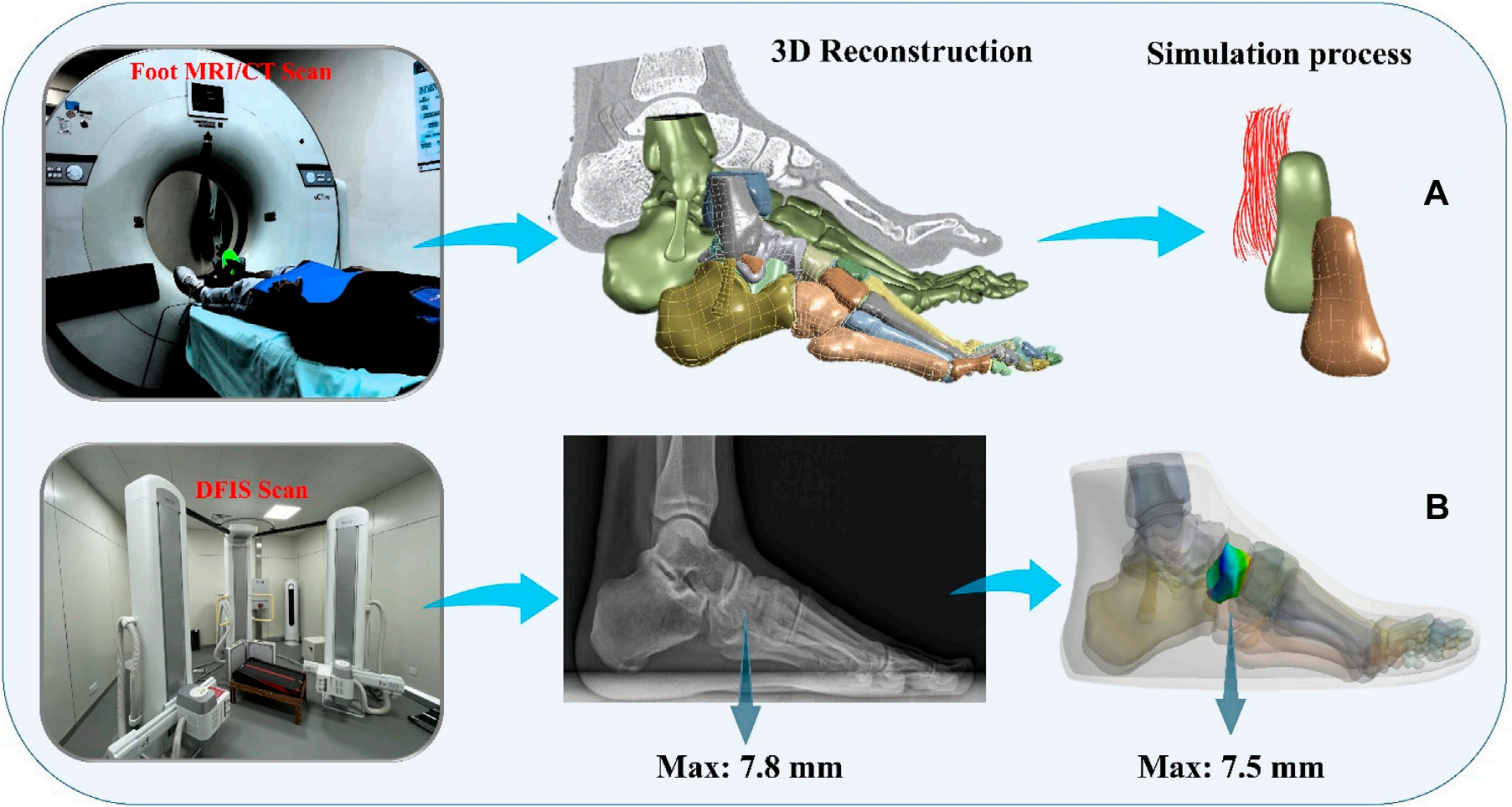
Finite element simulation workflows. **(A)** Composite representation of the anterior talofibular ligament; **(B)** Finite element model validation.

In the finite element model, the lateral collateral ligament (LAL) of the ankle was initially simulated too homogeneously, and this simplistic representation could not accurately capture the laxity of the lateral ankle ligament. Setting the lateral ankle ligament to be isotropic may lead to inaccurate results (Siegler et al., 1988). Therefore, we utilized a refined hyperelastic composite to model the physiological structure of the LAL, incorporating collagen fibers for enhanced accuracy (Figure 3A). This composite LAL model comprises a proteoglycan matrix reinforced by collagen fibers, with a collagen fiber volume fraction set at 60% (Kumai et al., 2002). This value was determined based on the upper limit of the total cross-sectional area occupied by collagenous proto-fibers in the LAL.

The strain energy formula for hyperelastic material is derived from the strain energy density function:

$$\Psi =\Psi_{vol}\left(J\right)+\Psi_{iso}^{m}\left(\bar{C}\right)+\Psi_{iso}^{f}\left(\;\lambda \right)$$ (1)

In this study, a Neo-Hookean model was employed. The volumetric component, denoted as $\Psi_{iso}$ , represents the change in ligament volume. Additionally, $\Psi_{iso}$ denotes the deviatoric component of shape change, where $\Psi_{iso}^{m}$ corresponds to the Stromal Fraction and $\Psi_{iso}^{f}$ represents the fiber fraction. J stands for the Jacques ratio of the deformation gradient tensor F , while $\bar{C}$ signifies the deviatoric component of the deformation gradient tensor C ( $\bar{C}=J^{-\frac{2}{3}}C$ ) (Weiss et al., 2002). Moreover, the attachment zones centers at both ends of the ligament served as the initial direction of the fiber. The elongation λ was computed based on the material’s deformation and the initial fiber direction $a_{0}$ ( $\lambda =\bar{C}\times a_{0}^{2}$ ).

For the volumetric component $\Psi_{vol}$ :

$$\Psi_{vol}\left(J\right)=\frac{1}{2D}\ln J^{2}$$ (2)

For the deviatoric component $\Psi_{iso}^{m}\left(\bar{C}\right)$ :

$$\Psi_{iso}^{m}\left(\bar{C}\right)=C_{1}\left(\bar{I_{1}}-3\right)$$ (3)

Limited ability of collagen fibers in the ligament to withstand loads, so it is defined as (Weiss et al., 1996):

$$\Psi_{iso}^{f}\left(\;\lambda \right)=F_{2}\left(\lambda \right)$$ (4)

Therefore, the strain energy equation is determined as:

$$\Psi =\frac{1}{2D}\ln J^{2}+C_{1}\left(\bar{I_{1}}-3\right)+F_{2}\left(\lambda \right)$$ (5)

### 3.4 Boundary, loading condition and model validation

To simulate the real situation, in this study, the angle of the ankle joint was determined based on the position of the foot model. This was achieved by fixing the floor and adjusting the angle between the tibial axis and the longitudinal axis of the foot in the sagittal plane within the finite element model. The adjustment ensured alignment between the global coordinate system and the original coordinate system of OpenSim (Delp et al., 2007). The inertial forces experienced during landing were simulated by applying ankle moments and reaction forces to the talar sliding joint and tibiotalar joint surfaces, respectively. Additionally, joint forces of the MPJ were applied to the upper surface of the first metatarsal and proximal phalanx bone. All materials were assumed to be isotropic and linearly elastic, except for the encapsulated soft tissues, skin, and ligaments, for which properties were derived from previous studies (Table 2). In this study, fluoroscopic image data of the ankle joints of subjects in the grounded condition were acquired using a high-speed dual fluoroscopic imaging system (DFIS) (Ti-WISH-II, Ti-Motion Ltd., Shanghai City, CN). These computed ankle joint displacements were then compared with the results obtained from finite-element analysis to validate its accuracy (Roach et al., 2017). Specifically, we validated the accuracy of the finite element model of the foot by measuring the displacement of the navicular bone, which is commonly used in clinical practice to assess specific deformations of the foot.

**TABLE 2:** Material properties of the components in the finite element model.

Component	Element type	Elastic modules (MPa): E	Poisson’s ratio: v	Destiny(kg/m^3^)	Reference
Skin	Tetrahedral solid	Hyperelastic (first-order Ogden model, μ=0.122 kPa,α=18 )	N/A	950	Pailler-Mattei et al. (2008)
Bulk soft tissue	Tetrahedral solid	Hyperelastic (second-order polynomial strain, $C_{10}=0.8556,C_{01}=0.05841,C_{20}=0.03900,C_{11}=0.02319,C_{02}=0.00851,D_{1}=3.65273$ )	N/A	950	Gu et al. (2010)
Bones	Tetrahedral solid	7,300	0.3	1,500	Cheung et al. (2005)
Cartilages	Tetrahedral solid	1	0.4	1,050	
Ligaments	Two-node truss	260	0.4	1,000	Kitaoka et al. (1994)
ATFL PTFL CFL	Tetrahedral solid	Hyperelastic (second-order polynomial strain, $C_{10}=-222.1,C_{01}=290.97,C_{20}=-1.1257,C_{11}=4.7267,C_{02}=79.602$	N/A	1,000	Peng et al. (2023)
Achilles Tendon	Two-node truss	816	0.3	1,000	Chen et al. (2012)
Plate	Hexahedral solid	17,000	0.4	1,000	Xiang et al. (2022)

ATFL, anterior talofibular ligament; PTFL, posterior talofibular ligament; CFL, calcaneofibular ligament.

First, high-resolution images of the ankle joint were obtained using DFIS imaging technology to ensure clarity and accuracy. These images were then imported into Rhinoceros software for detailed model adjustments. Next, the displacement of the navicular bone was calculated using the coordinate system calculator plugin in Rhinoceros (Li et al., 2008). This plugin accurately measures the spatial position changes of various points in the model to compute the actual displacement data of the navicular bone. Finally, these calculated results were compared with the finite element model for verification. The finite element model provides theoretical displacement data, and by comparing it with the actual calculated displacement data, the accuracy of the model can be validated (Figure 3B) (Nielsen et al., 2009; Koo and Li, 2016).

DFIS system generates high voltage through a high-voltage generator, and the X-ray tube is used to generate X-rays. The X-ray tube contains a cathode and an anode, when high voltage is applied to the X-ray tube, the cathode releases free electrons. These changes produce an image on the detector, forming an X-ray fluoroscopic image. The DFIS system is equipped with two X-ray sources and two detectors, which are positioned on each side of the object under observation. This setup allows for the simultaneous acquisition of X-ray fluoroscopic images from both directions, enabling reliable medical image data for clinical diagnosis.

## 4 Result

This study investigated the kinematic and kinetic changes in healthy individuals and patients with CLAI during landing. Additionally, we explored the stress distribution using a finite element model. The specific findings are detailed as follows:

### 4.1 Joint angle and moment

**FIGURE 4:**
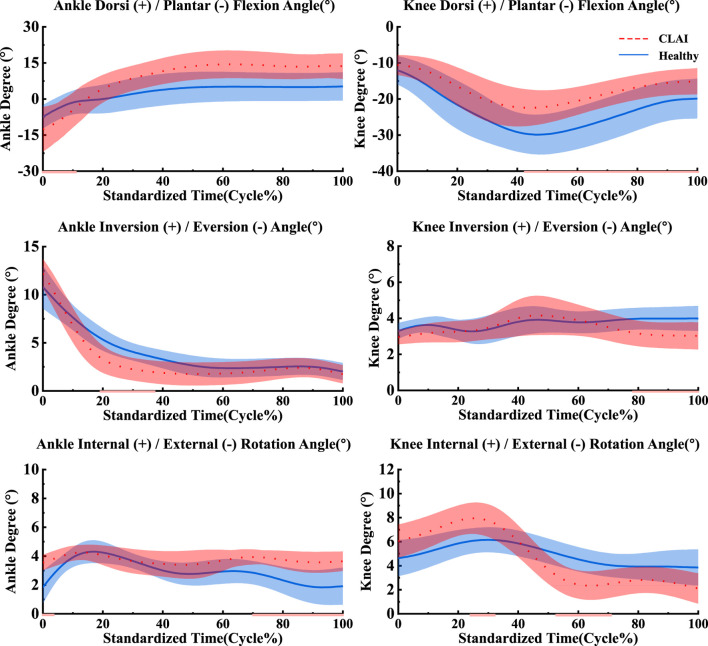
Mean and standard deviation of the ankle and knee joints. The red line illustrates the SPM analysis findings between the CLAI and healthy groups. From top to bottom are sagittal plane, coronal plane and horizontal plane.

According to Figure 4, during the 0%–14% landing phase, the ankle joint angle in the sagittal plane shows significant changes in CLAI patients compared to the healthy group (*p* < 0.001). In the coronal plane, during the 19%–38% landing phase, the ankle joint angle changes in the healthy group are significantly greater than those in CLAI patients (*p* = 0.033). In the horizontal plane, during the initial 0%–3% landing phase, the ankle joint angle changes in CLAI patients are significantly greater than in the healthy group (*p* = 0.016). Additionally, during the 70%–100% landing phase, the ankle joint angle in CLAI patients shows significant changes compared to the healthy group (*p* < 0.001).

For the knee joint, in the sagittal plane, CLAI patients are greater than that of the healthy group. Significant changes in the knee joint angle of CLAI patients are observed during the 42%–100% landing phase (*p* < 0.001). In the coronal plane, during the 77%–100% landing phase, the knee joint angle changes in the healthy group are significantly greater than those in CLAI patients (*p* < 0.05). In the horizontal plane, during the 23%–31% landing phase, the knee joint angle changes in CLAI patients are significantly greater than in the healthy group (*p* < 0.001), while during the 52%–71% phase, the angle changes are significantly smaller than in the healthy group (*p* < 0.05) (Table 3).

**TABLE 3:** Detailed biomechanical results between CLAI and Healthy group.

Parameters	CLAI	Healthy	P	ES
Sagittal plane peak ankle moment (Nm)	0.93	1.08	<0.01	0.274
Coronal plane peak ankle moment (Nm)	0.57	0.48	<0.05	0.162
Horizon plane peak ankle moment (Nm)	0.44	0.39	0.05	0.121
Sagittal plane peak knee moment (Nm)	1.87	1.69	<0.001	0.398
Coronal plane peak knee moment (Nm)	0.83	0.66	<0.001	0.285
Horizon plane peak knee moment (Nm)	0.61	0.55	<0.05	0.212
Sagittal plane peak ankle angle (°)	12.87	6.51	<0.001	0.863
Coronal plane peak ankle angle (°)	12.44	10.37	<0.05	0.195
Horizon plane peak ankle angle (°)	4.12	4.19	0.68	0.012
Sagittal plane peak knee angle (°)	−21.78	−29.95	<0.001	0.531
Coronal plane peak knee angle (°)	3.86	3.72	0.05	0.117
Horizon plane peak knee angle (°)	8.03	5.74	<0.001	0.416

ES, effect size; CLAI, chronic lateral ankle instability.

The results indicate that in the sagittal plane, the peak ankle joint moment in the CLAI group is significantly smaller than in the healthy group (*p* < 0.01). In contrast, in the coronal plane, the peak ankle joint moment in the CLAI group is significantly higher than in the healthy group (*p* < 0.05). However, in the horizon plane, there was no significant difference between the two groups (*p* = 0.05). For the knee joint moment in the sagittal plane, the CLAI group showed significantly higher compared to the healthy group (*p* < 0.001). Similarly, in the coronal plane, the knee joint moment was significantly greater in the CLAI group than in the healthy group (*p* < 0.01). Differences were also observed in the horizon plane, with the CLAI group exhibiting higher knee joint moments than the healthy group (*p* < 0.05) (Table 3).

### 4.2 Muscle activation

**FIGURE 5:**
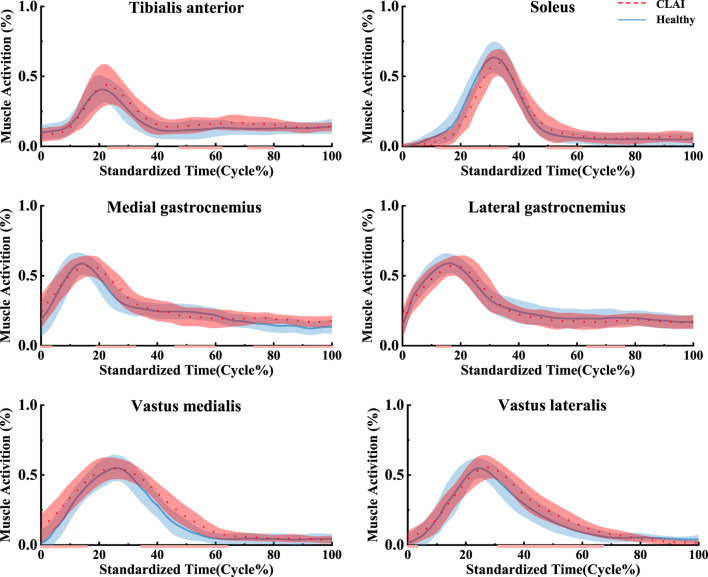
Mean and standard deviation of the simulated muscle activations. The red line illustrates the SPM analysis findings between the CLAI and healthy groups.

Patients with chronic lateral ankle instability (CLAI) exhibit different patterns of muscle activation compared to the healthy group during landing. Based on Figure 5, the muscle activation of the vastus lateralis muscle during landing was higher in CLAI patients compared to the healthy group (*p* < 0.05). Additionally, the results revealed that during the initial 0%–12% phase of landing, the muscle activation of the vastus medialis muscle in CLAI patients preceded that of the healthy group (*p* < 0.001), with higher peak muscle activation observed as well. However, injury to the lateral ankle ligament during landing resulted in significant differences. Conversely, during the 8%–35% landing phase (*p* = 0.012), the healthy group exhibited significantly higher muscle activation in the soleus compared to the CLAI group. Regarding the medial gastrocnemius and lateral gastrocnemius muscles, the results demonstrated a similar disparity in muscle activation between the healthy group and the CLAI group. Muscle activation in the medial gastrocnemius exhibited an earlier peak activation in the healthy group (*p* < 0.001), along with a higher peak muscle activation. Similarly, activation of the lateral gastrocnemius muscle occurred earlier in the healthy group compared to the CLAI group (*p* < 0.001). Furthermore, significant differences were observed in the tibialis anterior muscle between the CLAI and healthy groups during the 22%–40% and 48%–61% landing phases (*p* < 0.05).

### 4.3 Muscle force

**FIGURE 6:**
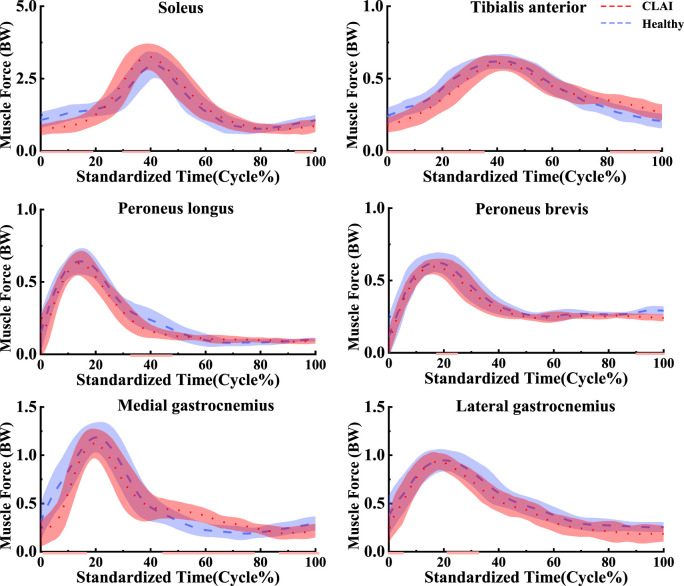
Mean and standard deviation of the simulated muscle forces. The red line illustrates the SPM analysis findings between the CLAI and healthy groups.

Based on SPM1d analysis, according to Figure 6, significant differences were observed in the muscle force of the tibialis anterior muscle between the healthy group and CLAI patients during the 0%–36% landing phase (*p* < 0.05). Conversely, during the 81%–98% phase of landing, the CLAI group exhibited higher muscle force compared to the healthy group. For the soleus, the CLAI group was higher than the healthy group most of the time, notably demonstrating a significant difference during the 30%–40% phase (*p* < 0.05). However, it was lower than the healthy group during the 0%–19% and 93%–100% landing phases (*p* < 0.001, *p* < 0.05, respectively). The muscle force of the peroneus longus muscle in the CLAI group was comparable to that of the healthy group, except for the 32%–44% phase of the landing, where it was lower than the healthy group (*p* < 0.001). Meanwhile, the medial gastrocnemius muscle force was significantly higher in the healthy group than in the CLAI group during the 0%–16% landing phase (*p* < 0.001), while in the 42%–78% phase (*p* < 0.001) it was higher in the CLAI group than in the healthy group. Lateral gastrocnemius muscle force was essentially similar between the two groups, although differences were observed during the 0%–5% and 21%–32% landing phases (*p* = 0.034, *p* < 0.05, respectively).

### 4.4 Stress distribution results

**FIGURE 7:**
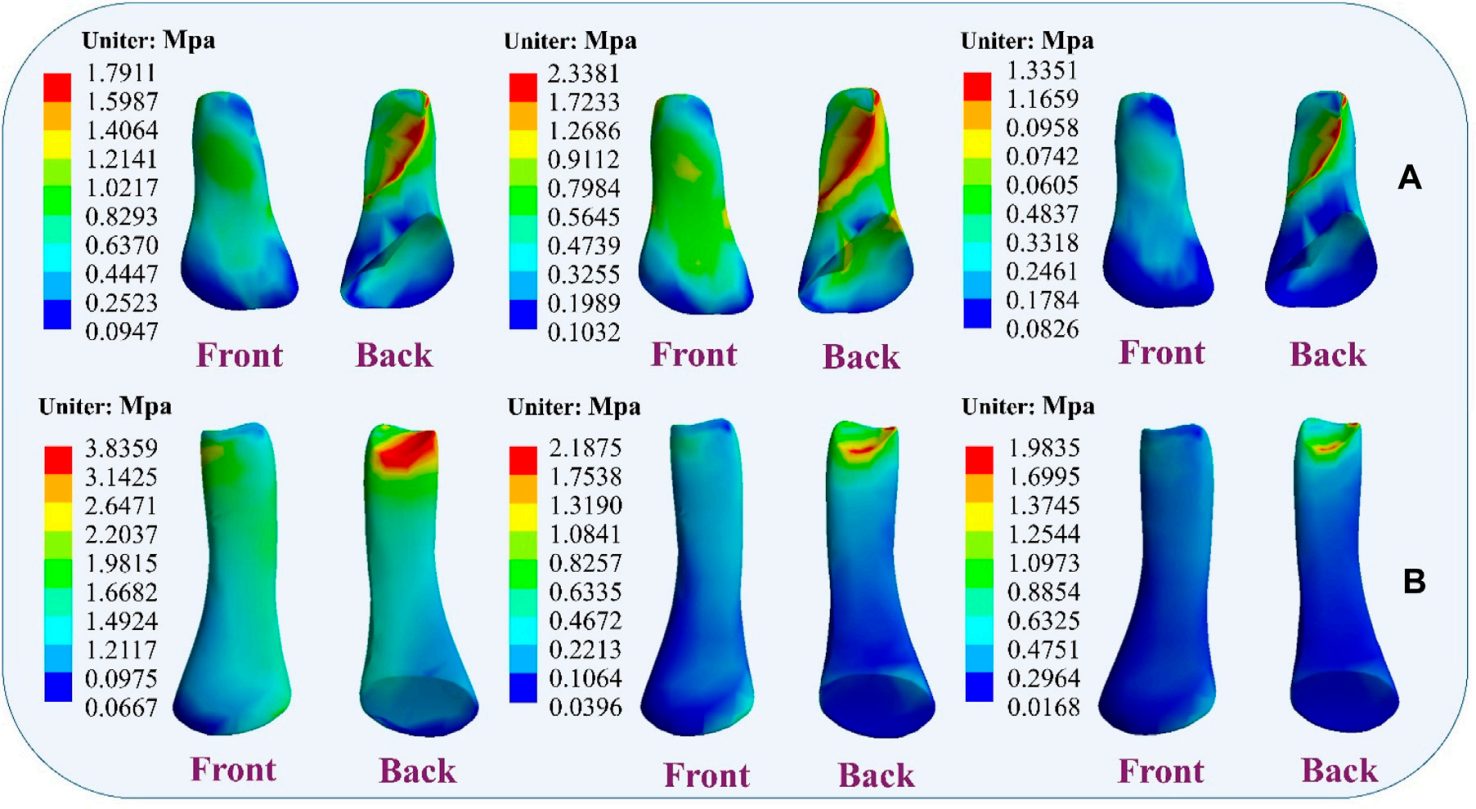
Distribution of ligament von Mises stress response during ligament laxity. The results from left to right are anterior talofibular ligament laxity, calcaneofibular ligament laxity, and both ligaments laxity. **(A)** anterior talofibular ligament; **(B)** calcaneofibular ligament.

**FIGURE 8:**
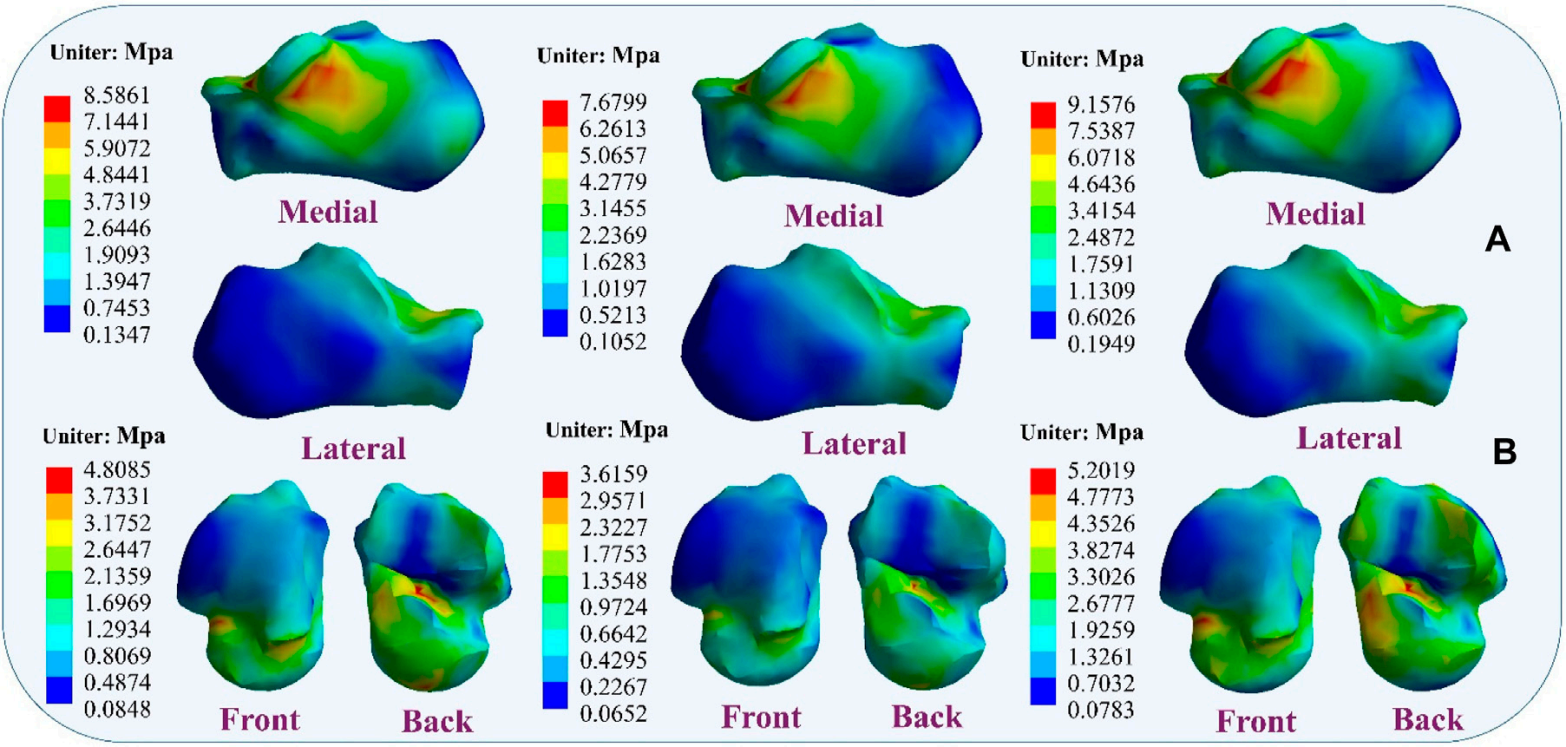
Distribution of von Mises stress response during ligament laxity. The results from left to right are anterior talofibular ligament laxity, calcaneofibular ligament laxity, and both ligaments laxity. **(A)** calcaneus bone; **(B)** talus bone.

During the simulation of a CLAI patient’s landing, the thermograms generated from finite element analysis illustrate changes in stress distribution on the ligaments as different ligaments exhibit laxity (Figure 7). When the ATFL exhibits laxity, the peak von Mises stress in the CFL rises to 3.8359 MPa, while the peak von Mises stress within the ATFL itself is 1.7911 MPa. A similar situation was observed when the CFL exhibited laxity. The stress on the ATFL increased significantly to 2.3381 MPa compared to its laxity. The CFL did not experience any additional stress and only 2.1875 MPa. However, some differences emerge when the ATFL and CFL exhibit laxity. The stresses experienced by the ATFL and CFL do not increase, with the peak von Mises stress on the ATFL being 1.3351 MPa, and on the CFL being 1.9835 MPa. Figure 8 illustrates the effect of ligament laxity on the calcaneus and talus bones. Under conditions where the CFL exhibits laxity, the peak von Mises stress on the calcaneus is 7.6799 MPa. However, when the ATFL exhibits laxity, the stress on the calcaneus increases to 8.5861 MPa, and the overall stress distribution is higher than when the CFL exhibits laxity. When both the ATFL and CFL exhibit laxity, the peak von Mises stress on the calcaneus significantly increases to 9.1576 MPa. The stress distribution of the talus bone shows a similar trend. When the ATFL exhibits laxity, the peak von Mises stress on the talus bone is 4.8085 MPa, while under CFL laxity, the stress on the talus bone is slightly lower at 3.6159 MPa. When both ligaments exhibit laxity, the peak von Mises stress on the talus bone increases to 5.2019 MPa.

**FIGURE 9:**
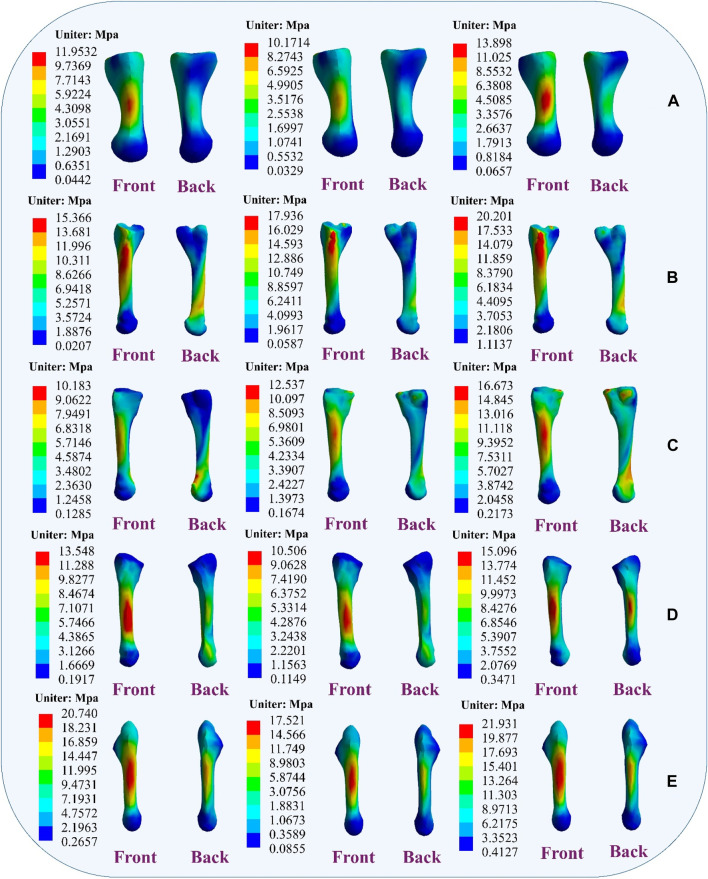
Distribution of metatarsal von Mises stress response during ligament laxity. The results from left to right are anterior talofibular ligament laxity, calcaneofibular ligament laxity and both ligaments laxity. **(A)** first metatarsal; **(B)** second metatarsal; **(C)** third metatarsal; **(D)** fourth metatarsal; **(E)** fifth metatarsal.

Figure 9 illustrates the changes in stress on different metatarsal bones when ligaments exhibit laxity. The stress variation is relatively small in the first metatarsal bone, with a peak von Mises stress of 11.9532 MPa observed when the ATFL exhibits laxity. Under CFL laxity conditions, the peak von Mises stress measures only 10.1714 MPa, slightly lower than observed with ATFL laxity. However, with simultaneous laxity in both ligaments, the peak von Mises stress reaches 13.898 MPa. However, the second metatarsal bone exhibits a different pattern. With the ATFL laxity, the peak von Mises stress measures 15.366 MPa, which increases to 17.936 MPa under CFL laxity. When laxity occurs in both ligaments simultaneously, the peak von Mises stress rises to 20.201 MPa. The third metatarsal bone demonstrates a behavior that resembles that of the second metatarsal bone, showing a peak von Mises stress of 10.183 MPa under ATFL laxity and 12.537 MPa under CFL laxity. With laxity present in both ligaments, the peak von Mises stress increases to 16.673 MPa. The variation in peak von Mises stress is less noticeable in the fourth metatarsal bone, with 13.548 MPa when the ATFL exhibits laxity, 10.506 MPa when the CFL exhibits laxity, and 15.096 MPa when both ligaments exhibit laxity. The fifth metatarsal bone exhibits the highest von Mises stress among the metatarsals. Under ATFL laxity, the peak von Mises stress measures 20.740 MPa, while under CFL laxity, it measures 17.521 MPa. Simultaneous laxity in both ligaments results in a peak von Mises stress of 21.931 MPa.

## 5 Discussion

The purpose of this study was to examine alterations in stress response and neural control during landing among patients with lateral collateral ligament injuries of the ankle. We conducted a comparative analysis of metatarsal stress changes during landing in subjects exhibiting varying degrees of lateral collateral ligament laxity in the ankle, aiming to enhance ankle stability in patients. Additionally, we aimed to contribute to the development of more scientifically informed treatment protocols for the rehabilitation of such patients through further research. We hypothesized that metatarsal stress would vary with the degree of laxity of the lateral ligament of the ankle joint, thus affecting the stability of the ankle joint. The results of this study are consistent with our initial hypothesis.

The study found that compared to the healthy group, CLAI patients exhibited greater ankle joint angle changes in the sagittal plane. This difference may be due to their inability to effectively regulate ankle joint angles upon landing, leading to abnormal joint movement patterns. Additionally, there were differences in knee joint angles between the healthy group and CLAI patients, indicating distinct knee joint movement control strategies between the two groups. These findings are consistent with previous research, suggesting that CLAI patients display altered landing patterns compared to the healthy group (Brown et al., 2008). Gehring et al. (2014) research highlights significant deviations in knee joint angles during landing and cutting movements in the CLAI group compared to the healthy group, emphasizing the importance of considering unanticipated tasks when assessing actual LAL. The study revealed that the CLAI group employed different movement strategies during landing tasks than the healthy group. Further research is needed to verify the effectiveness of the observed changes in neural control strategies in reducing the risk of LAL.

This study observed that laxity of the lateral ankle ligaments increases stress on the metatarsals, leading to ankle instability. Research indicates that damage or laxity of the lateral ligaments alters the biomechanical properties of the ankle joint. Typically, the lateral ankle ligaments maintain stability by supporting and restricting joint movement (Bonnel et al., 2010; Jiang et al., 2024). Damage to these ligaments may result in increased pressure on the metatarsals, impairing their ability to effectively support ankle movement. The findings also corroborate previous research, showing that further laxity of the ATFL and CFL significantly alters metatarsal stress during simulated CLAI landings. Under CFL laxity, peak von Mises stress on the fifth metatarsal increases, and under ATFL laxity, the peak von Mises stress on the fifth metatarsal is even greater, reaching its peak when both ligaments are lax. Studies by Doherty indicate that lateral ankle ligament injuries restrict inversion and eversion movements of the ankle joint, leading to additional load on the metatarsals and increasing the risk of ankle injury (Doherty et al., 2016). Dobbe et al. observed increased metatarsal stress in patients with chronic lateral ankle instability, particularly during physical activity (Dobbe et al., 2020). Additionally, the study results demonstrate that ligament stress gradually increases upon landing with lateral ligament laxity, suggesting changes in the surrounding tissues and structures, consistent with previous findings. During this process, the mechanical properties of the ligaments may change, making them more sensitive to external stress and potentially increasing the risk of secondary injury when damaged ligaments are subjected to external stress (Barelds et al., 2018).

Furthermore, the study found that ligament laxity can induce muscle compensation. Results suggest that laxity of the ATFL may transfer stress to the CFL, and conversely, laxity of the CFL may increase stress on the ATFL. When two ligaments are lax, stress may transfer to other muscles to maintain the stability of the foot structure. This muscle compensation may involve muscles and ligaments surrounding the foot. These findings are significant for understanding muscle strength reconstruction during ligament injury rehabilitation and preventing further injury. Banerjee and Agarwal. (1998) research emphasizes the indispensable role of the talus and calcaneus in weight-bearing, providing anchorage for other crucial ligament structures, thereby tightly connecting the critical joints of the distal lower limb. Laxity of the lateral ankle ligaments may further damage the talus and calcaneus. Moreover, the study also found that with ligament laxity, stress between the calcaneus and talus changes, potentially leading to talus displacement and affecting joint stability. Therefore, maintaining the stability of the lateral ankle ligaments is crucial for protecting the talus and calcaneus and preserving the function and stability of the foot joints (Ferran et al., 2009; Kang and Jiang, 2024).

Another noteworthy discovery in the present study is the distinct muscle activation pattern observed in patients with chronic lateral ankle instability (CLAI) during landing compared to the healthy group, which corroborates earlier research. DeJong et al. (2020) demonstrated that individuals with CLAI showcased compensatory muscle activation in the proximal joint, while Rios et al. (2015) observed heightened muscle activation in CLAI patients during single-leg stance compared to healthy controls, particularly in the activation of proximal joint muscles during the standing phase. The current study noted increased muscle activation in the medial and lateral femoral muscles among CLAI patients compared to the healthy group. This indicates that individuals with CLAI tend to rely on heightened proximal muscle activation as a compensatory mechanism to improve motor control and mitigate neuromuscular deficits in the ankle joint. Previous research has similarly highlighted decreased neuromuscular recruitment as a common manifestation following LAL injury (Feger et al., 2014). The ligament injury may lead to decreased joint positional sensation, thereby reducing muscle sensitivity and responsiveness to nerve signals. The study findings indicated delayed activation of the medial gastrocnemius and lateral gastrocnemius muscles in the CLAI group compared to the healthy group. Furthermore, the soleus muscle exhibited not only earlier activation but also higher peak muscle activation. H. Kim’s analysis revealed that muscles like the peroneus longus, medial gastrocnemius, and lateral gastrocnemius were activated earlier during walking in the healthy group. Conversely, CLAI patients displayed delayed activation of the lateral gastrocnemius and soleus muscles during landing, potentially contributing to ankle instability (Kim et al., 2019). Moreover, CLAI patients exhibited greater tibialis anterior muscle strength and activation compared to the healthy group, indicating an augmented reliance on ankle dorsiflexion for stabilization during landing (Jie et al., 2024). The above study suggests that CLAI patients exhibit altered motor control patterns during landing compared to the healthy group. In the absence of adequate treatment and rehabilitation, CLAI patients may employ alternative strategies to compensate for ankle instability, potentially heightening the risk of secondary injury (Son et al., 2019).

Given the observed compensatory muscle activation patterns in patients with CLAI, targeted rehabilitation programs can be developed to address these neuromuscular deficits. For instance, strengthening exercises focused on the medial and lateral femoral muscles may help improve proximal muscle support and overall joint stability (Xu et al., 2022; Zhou and Ugbolue, 2024). Additionally, proprioceptive training can be incorporated to enhance joint positional sense and muscle responsiveness, thereby reducing the risk of further injury. By customizing rehabilitation protocols to address specific muscle activation delays and deficiencies identified in this study, doctors can optimize recovery and functional outcomes for individuals with CLAI (Watabe et al., 2021). Such strategies not only aim to restore normal muscle activation patterns but also to prevent compensatory mechanisms that could lead to secondary injuries.

This study also has several limitations. Firstly, only one patient with stabilized chronic lateral ankle instability was included in the development of the finite element model. Given inherent individual differences, the conclusions drawn from the study may vary. Additionally, while the ligaments were modeled as hyperelastic in this study, the overall rigidity of the model was only tailored to a single individual and did not fully encompass collective variations. Moreover, the boundary conditions of the models were uniform and did not simulate the actual process of landing injury. Variations in setup conditions such as material properties, mesh size, and mesh behavior could significantly influence the results. It’s important to acknowledge that this scenario may not entirely reflect real-world conditions. Meanwhile, only displacement of navicular height was used in the model validation. Subsequent experiments should include validation through plantar pressure distribution, joint contact stress, and contact area.

## 6 Conclusion

In this study, we observed the impact of lateral ankle ligament laxity on the biomechanical characteristics and stability of the foot. The results indicate that injury or laxity of the lateral ligaments may lead to increased stress on the metatarsals, thereby compromising the stability of the ankle joint. Specifically, further laxity of the ATFL and CFL significantly altered the distribution of stress on the metatarsals, resulting in a higher risk of ankle injury. Additionally, we observed that ligament laxity may trigger muscle compensation, further affecting the stability of the foot structure. These findings are crucial for understanding the rebuilding of muscle strength during the rehabilitation process following ligament injuries and for preventing recurrent injuries. Therefore, maintaining the stability of the lateral ankle ligaments is essential for preserving the function and stability of the foot joints.

## Data Availability

The original contributions presented in the study are included in the article/Supplementary Material, further inquiries can be directed to the corresponding authors.
